# Blockchain Technology in Healthcare: A Systematic Review

**DOI:** 10.3390/healthcare7020056

**Published:** 2019-04-04

**Authors:** Cornelius C. Agbo, Qusay H. Mahmoud, J. Mikael Eklund

**Affiliations:** Department of Electrical, Computer and Software Engineering, University of Ontario Institute of Technology, Oshawa, ON L1G 0C5, Canada; qusay.mahmoud@uoit.ca (Q.H.M.); mikael.eklund@uoit.ca (J.M.E.)

**Keywords:** blockchain, healthcare, systematic review

## Abstract

Since blockchain was introduced through Bitcoin, research has been ongoing to extend its applications to non-financial use cases. Healthcare is one industry in which blockchain is expected to have significant impacts. Research in this area is relatively new but growing rapidly; so, health informatics researchers and practitioners are always struggling to keep pace with research progress in this area. This paper reports on a systematic review of the ongoing research in the application of blockchain technology in healthcare. The research methodology is based on the Preferred Reporting Items for Systematic Reviews and Meta-Analysis (PRISMA) guidelines and a systematic mapping study process, in which a well-designed search protocol is used to search four scientific databases, to identify, extract and analyze all relevant publications. The review shows that a number of studies have proposed different use cases for the application of blockchain in healthcare; however, there is a lack of adequate prototype implementations and studies to characterize the effectiveness of these proposed use cases. The review further highlights the state-of-the-art in the development of blockchain applications for healthcare, their limitations and the areas for future research. To this end, therefore, there is still the need for more research to better understand, characterize and evaluate the utility of blockchain in healthcare.

## 1. Introduction

Blockchain gained popularity as a distributed ledger technology following the Bitcoin white paper published in October, 2008 [1]. As the underlying technology for Bitcoin, the main utility of blockchain is that it makes possible the exchange of electronic coins among participants in a distributed network without the need for a centralized, trusted third party. Transactions involving the exchange of electronic currencies between persons or companies have traditionally relied on a trusted third party (TTP), such as a bank, as a mediator. The reliance on a TTP is not desirable for a number of reasons. A trusted third party may malfunction, fail or be compromised maliciously to render the financial system unavailable or insecure; thus, a TTP undermines a system potentially as a single point of failure. A TTP also charges transaction fees and adds some transaction delays. The motivation behind Bitcoins is, therefore, to overcome these limitations associated with the reliance on TTP in electronic transactions. 

A year after the publication of the famous white paper on Bitcoin, the Bitcoin cryptocurrency was implemented, with the code released as open-source, which made it possible for others to modify the code and improve on it to create different generations of blockchain-based technologies. The first implementations of blockchain-based cryptocurrencies, such as the Bitcoin, constitute the first generation of blockchain technology, which is also referred to as blockchain 1.0 [2]. Other blockchain 1.0 technologies include Monero [3], Dash [4] and Litecoin [5], to name a few. The second generation of blockchain technology (blockchain 2.0) is associated with the introduction of smart properties and smart contracts [2]. The smart properties are those digital properties or assets whose ownership can be controlled by a blockchain-based platform, while the smart contracts are the software programs that encode the rules of how the smart properties are controlled and managed. Examples of blockchain 2.0 cryptocurrencies include Ethereum [6], Ethereum Classic [7], NEO [8] and QTUM [9]. 

Building on the above, the third generation of blockchain technology (blockchain 3.0) is now concerned with the non-financial applications of blockchain [2]. To this end, efforts have been made to adapt the technology to other application areas, outside finance, so that other industries and use cases can benefit from the interesting features of blockchain. Consequently, blockchain is now considered as a general purpose technology [10,11] that has found applications in different industries and use cases, such as identity management, dispute resolution, contract management, supply chain management, insurance and healthcare, to name a few [10,12].

With the growing fascination for blockchain and its adoption in different organizations and industries, healthcare has come to represent a significant area where a number of use cases have been identified for the application of blockchain. However, blockchain being a relatively new technology and with a lot of hype in the press as well as in grey publications in the form of opinion pieces, commentaries, blog posts, interviews, etc., there is a lot of inaccurate information, speculations and uncertainties about the potential utility of blockchain in the healthcare industry. Members of the research community and practitioners would want to understand the specific areas of application or use cases of blockchain in the healthcare industry; and of these identified use cases, what blockchain-based healthcare applications have been developed? What are the challenges and limitations of the blockchain-based healthcare applications, how are these challenges currently being addressed and what are the areas for improvement? 

This paper reports on the systematic review that is conducted to address the above questions. While there exist some interesting reviews in the literature that are related to this topic [13,14,15,16,17], ours is different in terms of the methodology and the objectives. In the review conducted by Angraal et al. [13], they identify some examples of the application of blockchain technology in healthcare. These include the Guardtime, a firm which operates a blockchain-based healthcare platform for the validation of patients’ identities for the citizens of Estonia; and the MedRec project, which was created to facilitate the management of permissions, authorization and data sharing between healthcare entities. Similarly, Engelhardt [14] outlines a collection of ‘noteworthy’ examples of blockchain technology companies in the healthcare sector. These companies are grouped under different healthcare use cases, namely; prescription drug fraud detection, patient-centered medical records and the dental industry. This review is equally similar to the one conducted by Mettler [15] where he reports some examples of blockchain-based applications and companies in the areas of public health management, medical research and drug counterfeiting in the pharmaceutical industry. On their part, Ku et al. [16] publishes the key benefits of blockchain when compared to traditional databases for healthcare applications. They go further to explain how these benefits can be harnessed to improve medical record management, enhance insurance claim processes, improve clinical research and advance healthcare data ledgers. Lastly, Roman-Belmonte et al. [17] in their review, cover the existing and potential applications of blockchain in different fields of medicine, which include the fields of legal medicine, health data analytics, biomedical research, electronic medical records, meaningful use, payment for medical services and so on. 

Our approach departs from the ones adopted in the aforementioned reviews in that we follow the guidelines for systematic literature review and systematic mapping study process [18,19] and the Preferred Reporting Items for Systematic Reviews and Meta-Analysis (PRISMA) statement [20] in conducting and reporting our review. Our systematic review is based on a well-designed research protocol that ensures a holistic and unbiased sampling of all the published peer-reviewed articles that are relevant to the subject matter. Based on this protocol, we retrieve from reputable scientific databases the relevant articles which we classify and map into different categories to unravel the true state of the ongoing research in the application of blockchain technology in healthcare. The resulting map from our research will be very valuable to practitioners and researchers in understanding the domain state-of-the-art and the areas for future research. To the best of our knowledge, this is the first literature review on the application of blockchain in healthcare that follows the systematic mapping study process. The systematic review conducted by Yli-Huumo et al. inspired our choice of this methodology; however, their review on the technical challenges of blockchain technology [21] is unrelated to our topic, which is the application of blockchain in healthcare. The systematic review by Holbl et al. [22] is also similar, but ours differs markedly in scope; for example, the review covers 33 articles, while ours covers 65 articles.

It is also worth noting that the objective of this review is not just to identify the use cases or the examples of blockchain-based applications in healthcare, but also to understand the limitations and challenges for the blockchain-based healthcare applications as well as the current trends in terms of the technical approaches, methodologies and concepts employed in developing these applications (and in overcoming the limitations) in a view to unravel the areas for future research. Moreover, this review covers many new materials which had not been published at the time of the previous reviews. As noted earlier, the application of blockchain in healthcare is a relatively new paradigm which is growing rapidly, and as such, there are many new publications on the topic. To illustrate this point, 32 of the 65 selected papers for this study were published in 2018 whereas most of the existing reviews were published in 2017 or earlier. 

The remainder of this paper is organized as follows. Section 2 provides a brief technical overview of blockchain and its application in healthcare. Section 3 describes the applied research methodology. Section 4, Section 5 and Section 6 present the results, the discussion and the conclusion, respectively.

## 2. Overview of Blockchain

The detailed technical underpinnings of the blockchain technology is outside the scope of this paper. However, for the purpose of our discussion going forward, it is important to shed light on some blockchain concepts, features and terminologies that will foster the understanding of how blockchain is applied to solve healthcare problems.

**Figure 1:**
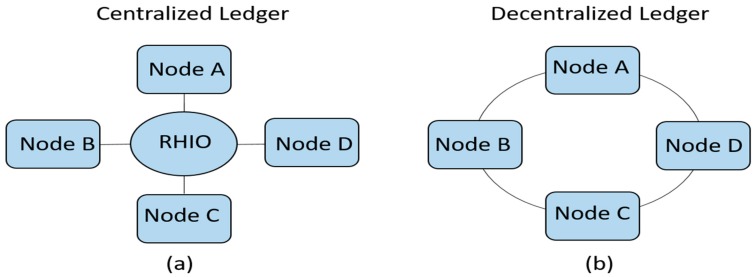
Centralized vs. decentralized system. In (**a**), there are multiple ledgers but all records are held in RHIO, whereas in (**b**), there is only one ledger but every node has some level of access to that ledger. The decentralized architecture removes the need for trusted third party, makes transactions faster, and removes the transaction fees charged by the trusted third party (RHIO).

Perhaps, the most obvious and outstanding benefit of blockchain is the fact that it removes the need for a centralized trusted third party in distributed applications. By making it possible for two or more parties to carry out transactions in a distributed environment without a centralized authority, blockchain overcomes the problem of single point of failure which a central authority would otherwise introduce. It also improves transaction speed, by removing the delay introduced by the central authority, and at the same time, it makes transactions cheaper since the transaction fees charged by the central authority is removed. In place of a central authority, blockchain uses a consensus mechanism to reconcile discrepancies between nodes in a distributed application. The difference between centralized and decentralized systems is illustrated in Figure 1. 

In Figure 1a, there are multiple ledgers but all the records are held in one central place, in this case, the Regional Health Information Organization (RHIO). In essence, the RHIO maintains the state of the ledger. When there is a disagreement between two nodes about the “true state” of the ledger, the RHIO is consulted as the final arbiter to determine the “true state” of the ledger. On the contrary, in Figure 1b, there is only one ledger, but all the nodes have a copy of the ledger and some level of access to its contents. To maintain the integrity of the ledger, the nodes must have a means to agree on the “true state” of the ledger, in the absence of a central authority. When the nodes agree on a particular “true state” of the ledger, it is referred to as consensus. The different ways in which consensus is achieved in blockchain will be explained in the remaining part of this Section. 

At its very core, blockchain uses cryptographic primitives to derive most of its capabilities. Participants in a blockchain network are represented as nodes and each node uses public key infrastructure (PKI) [23] to create and propose transactions. Each participant possesses a pair of public and private keys. The public key serves as the public address of the user while the private key is used to authenticate the user. When a transaction is created, it has to include the public key of the user who created the transaction, the public key of the receiver of the transaction and the transaction message. All of these are bundled together and cryptographically signed using the user’s private key and subsequently broadcast to the other nodes in the blockchain network. When this is done, the user is said to have proposed a transaction. 

A block is a collection of valid transaction proposals that are received within a period of time, say 10 min [1]. A valid transaction proposal is one which satisfies the validation requirements. The process of validation ensures that the proposed transaction is legitimate, for example, that it originates from an authorized user (node). The consensus algorithm determines the order in which the validated blocks are appended to the ledger. There are special nodes in a blockchain network that are responsible for running the consensus algorithms (i.e., for validating transactions and determining the order in which transaction blocks are added to the blockchain). These special nodes are called miners and the process of validating transactions and ordering them in the blockchain is referred to as mining. Once a transaction proposal is received by a miner, the miner proceeds to check if the transaction is valid. Validated transactions are included into a block. After a period (or block) of time, the new block of validated transactions is linked (or chained) to the previous blocks, creating a chain of blocks, known as blockchain. The blockchain is replicated among all the nodes in the network, such that every node has an identical database or ledger of all the transactions in the network.

It is important to understand how blocks are chained to form the blockchain, but let us first explain the different types of blockchain. In some implementations of blockchain, e.g., Bitcoin, any node is free to join the network and to participate in the mining process (that is, take part in validating new blocks and chaining them to the existing ones). This sort of blockchain implementation in which any node is free to join the network and to participate as a miner without requiring any authorization or access permission is referred to as public or permissionless blockchain. In contrast, permissioned blockchain is one in which participants must be authorized and have the right access permissions before they can join and participate in the network. In permissioned blockchain, only certain nodes may be permitted to participate in the mining process. By virtue of their characteristics, permissioned blockchain networks are more likely to be smaller, faster and more secure than the public blockchain networks. 

A permissioned blockchain may further be classified as a private or a consortium blockchain. The distinction between private and consortium blockchains is based on the number of nodes permitted to be miners. If only one node is permitted to be a miner, it is more aptly referred to as private blockchain. Note, however, that when only one node is the miner, then that node serves more or less as a central authority, in which case some of the advantages of decentralization is lost. Consortium blockchain is one in which two or more nodes are permitted to take part in the mining process, yet the blockchain network remains permissioned, in the sense that only authorized users can be part of the network. The consortium blockchain, therefore, has the advantages of decentralization as well as the improved security and privacy inherent in the private blockchain. More information about the different types of blockchain (public, permissioned: private and consortium) can be found in [24]. 

**Figure 2:**
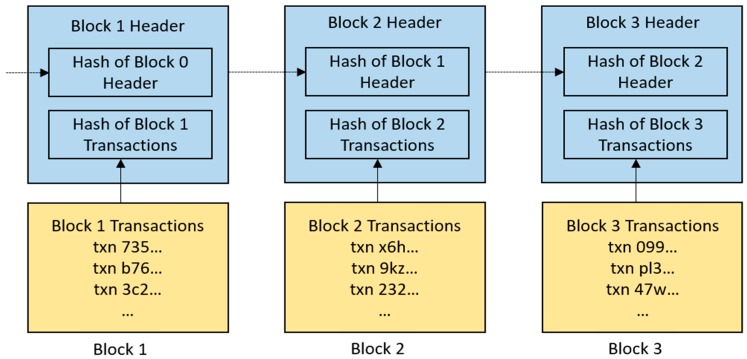
A simplified example of how blocks are chained to form a blockchain. Notice that each block contains a header and a number of transactions. The transactions in a block are hashed to generate a fixed-length hash output which is added to the block header. After the creation of the first block, every subsequent valid block must contain the hash output of the previous block header. The hash of the previous block header which is contained in every block serves as the chain that links every valid block to the ones before it. Thus, by linking every block to the previous blocks, a chain of blocks (blockchain) is established.

Let us get back to how blocks are chained to form a blockchain. The chaining of blocks is achieved through another cryptographic primitive which involves the use of hash functions. A hash function takes a message of arbitrary length and crunches it into a hash output of a fixed length, referred to as a message digest or a digital fingerprint. An interesting property of hash function is that it is collision-resistant, i.e., no two different messages will produce the same hash output. This property is the basis of block chaining. To chain a new block to the blockchain, the hash of the previous block header is included in the new block header. Thus, the last block in the blockchain contains the fingerprint of the transactions in the previous block, which in turn contains the fingerprint of the transactions in the preceding block and so on (Figure 2). 

As depicted in the diagram in Figure 2, if any of the transactions in a block is changed, even if slightly, the corresponding hash output will change drastically, which will break the chain to the subsequent blocks in the blockchain. Therefore, any alteration to the contents of a block in the blockchain is easily detected in the network. For this reason, once a transaction is added to a block and chained to the blockchain, that transaction cannot be altered or undone. Thus, information on the blockchain are said to be immutable. Immutability is an important property of blockchain which ensures that records, once created, cannot be retrieved or modified. To update a record on the blockchain, a new record must be created, hence, blockchain is also said to be an append-only ledger. The process of chaining blocks to the blockchain also ensures that the transactions are time-stamped, thereby creating an audit-trail of who did what and when.

As explained previously, the approaches to mining and reaching consensus differ across the different types of blockchain. In public blockchain, such as Bitcoin, there are usually more than one miner in the blockchain network, with the miners potentially receiving the transaction proposals in varying order, so it is possible that different miners will produce different valid blocks in which the ordering of transactions are different. In order for every node to have an identical copy of the blockchain, miners in the network must agree on the order in which the validated blocks are appended to the blockchain. In other words, only one of the miners is allowed to append a valid block per cycle. This can be done in different ways according to the consensus protocol in use. A popular example of a consensus protocol is the Proof of Work (PoW) used in Bitcoin [1].

Proof of Work (PoW) is a protocol based also on cryptographic hash function, in which the miners are required to solve a computationally difficult problem to determine the miner whose block is accepted to be added to the blockchain. In PoW protocol, a predetermined pattern of digital fingerprint is given, and the miners are required to find a random number which can be added to the transaction messages and hashed together to produce an identical pattern to the one given. In each cycle, the first miner to finish solving the mathematical problem is allowed to add a block to the blockchain. There are other examples of consensus protocols in the wild but their main purpose is the same, which is to ensure a consistent “true state” of the ledgers in the distributed nodes, without relying on a centralized trusted third party. 

From the foregoing, blockchain can be defined as an immutable ledger or database, shared by peers in a network, in which records of events or transactions are appended in a chronological order. Evidently, blockchain embodies some interesting features that are beneficial to healthcare applications.

One important feature of blockchain that is clearly beneficial to healthcare applications is decentralization which makes it possible to implement distributed healthcare applications that do not rely on a centralized authority. Additionally, the fact that the information in the blockchain is replicated among all the nodes in the network creates an atmosphere of transparency and openness, allowing healthcare stakeholders, and in particular the patients, to know how their data is used, by whom, when and how. More importantly, compromising any one node in the blockchain network does not affect the state of the ledger since the information in the ledger is replicated among multiple nodes in the network. Therefore, by its nature, blockchain can protect healthcare data from potential data loss, corruption or security attacks, such as the ransomware attack [25].

In addition, the immutability property of blockchain which makes it impossible to alter or modify any record that has been appended to the blockchain aligns very well with the requirements for storing healthcare records—it is very important to ensure the integrity and validity of patients’ health records. What is more, the use of cryptographic algorithms to encrypt the data stored on the blockchain ensures that only the users who have legitimate permissions to access the data can decrypt them, thereby improving the data security and privacy. Furthermore, since the identities of the patients in a blockchain are pseudonymized through the use of cryptographic keys, the health data of patients may be shared among healthcare stakeholders without revealing the identities of the patients. Blockchain also supports smart contracts [2] that can be used to program the rules that allow the patients to be in control of how their health records are shared or used. This is particularly relevant to the European General Data Protection Regulation (GDPR) which prohibits the processing of sensitive personal data of patients unless explicit consent is given, or specific conditions are met [26]. Therefore, blockchain can facilitate the development of a GPDR-compliant EMR management system, by encoding in the smart contract a set of rules that ensure that patients’ sensitive data cannot be shared or used without appropriate authorizations. The potential benefits of blockchain to healthcare applications are summed up in Table 1.

## 3. Research Methodology

**Figure 3:**
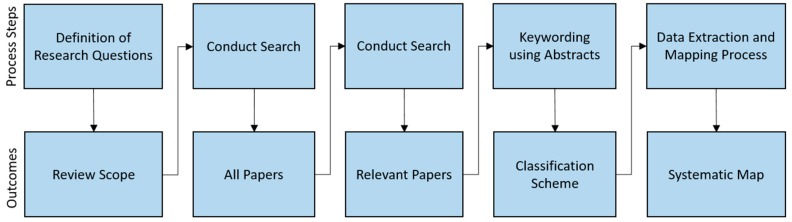
The systematic mapping process steps.

In conducting and reporting this review, we adopted the guidelines for systematic literature review [18] and the process for systematic mapping study [19], as well as the guidelines described in the PRISMA statement [20]. As explained in [19], the goal of a systematic mapping study is to get an overview of the research area, and to complement this by investigating the state of evidence in specific topics. In this case, the results of the mapping study would help us to identify and map the blockchain use cases in healthcare, and to understand the extent to which blockchain-based applications have been developed in relation to the identified use cases. They would also help us to identify areas of possible research gaps. The systematic review would again enable us to investigate the current trends in terms of the technical approaches, methodologies and concepts employed in developing blockchain-based healthcare applications. In what follows, we go through the systematic mapping process as shown in Figure 3. 

### 3.1. Definition of Research Questions

As the first process step in the systematic mapping study, we defined the following four research questions in line with our objective which is to unravel the state-of-the-art in research on the application of blockchain technology in healthcare.

#### 3.1.1. What Are the Use Cases of Blockchain in Healthcare?

The primary question in this research is to understand the different areas of healthcare that blockchain has been shown to find application. By reviewing the relevant articles from scientific databases, we are be able to identify what healthcare problems that blockchain can solve, and by so doing, isolate those problems which are better solved using other techniques. Given the frenzy in the media in which a lot of problems are deemed solvable by blockchain, a map of problem domains in healthcare in which blockchains are applicable will help researchers and practitioners to focus their interest on those promising areas of blockchain application in the industry.

#### 3.1.2. Of the Identified Use Cases, What Blockchain-Based Applications Have Been Developed?

Many areas of application of blockchain have been proposed in scientific literature. However, not all of these proposals have been translated into working prototypes. It is therefore important to understand the extent of real-world implementations of blockchain-based healthcare applications in relation to the identified use cases. This will help to highlight areas where there are research gaps and the need to shift research focus to these areas.

#### 3.1.3. What Are the Challenges and Limitations of the Blockchain-Based Applications?

This question seeks to understand the challenges facing the implementations of blockchain-based healthcare applications. Based on the prototype applications that have been developed, what are the limitations of this new technology in meeting the expected goals of solving healthcare-related problems?

#### 3.1.4. How Are These Challenges and Limitations Currently Being Addressed?

This research question seeks to understand the approaches taken to develop blockchain-based healthcare applications with a view to guiding future projects, so that there will be no need to reinvent the wheels. Since the first blockchain implementation in Bitcoin cryptocurrency, several modifications and improvements have been made to the technology to make it adaptable to non-financial use cases. This research question looks at the current trends in terms of the technical approaches and methodologies that are employed in developing blockchain-based applications for healthcare.

#### 3.1.5. What Are the Open Research Issues and the Areas for Future Research?

The last question addresses the issues for future research. Identifying research gaps and the challenges in the field will help researchers to streamline their future research to focus on addressing these research gaps and challenges.

### 3.2. Conducting the Research

In the second process step for systematic mapping study, the primary papers for the study are selected by searching the scientific databases using a search string or keywords. Four scientific databases were included in our search, namely; PubMed, IEEE Xplore, Web of Science and Scopus. By choosing these databases, our intention was to focus only on peer-reviewed articles that have been published in reputable journals, conferences, workshops, books or symposiums. To search the databases, we used the following search string: “blockchain” AND (“health*” OR “medic*” OR “biomedic*” OR “clinic*”). This selection of search string is based on pilot searches in which we tested some commonly used healthcare-related terms and acronyms, such as medical, biomedical, clinical, healthcare, etc., as well as EHR (Electronic Health Records), EMR (Electronic Medical Records) and PHR (Personal Health Records). It was observed that all of these healthcare-related terms and their derivatives as well as the acronyms are all captured in our search string as none of them when combined with “blockchain” returned any new result that is not returned with our search string. We also noticed that using only “blockchain” and “health*” does not retrieve some papers that do not have “health*”-related keywords in their metadata but may have used other health-related terms such as medical, biomedical or clinical. Searches with alternative strings to “blockchain,” such as “distributed ledger technology” did not return any new results. 

It is also important to point out that the literature search was conducted without any time restrictions, considering that the topic is relatively new, and therefore, all existing literature in this area were considered relevant to our study. 

### 3.3. Screening of Relevant Papers

After retrieving the papers from the databases based on our search protocol, the next step was to screen them for relevance. The first phase of this process step was to screen the papers for relevance based on their titles. Retrieved papers whose titles clearly indicate that they are not relevant to our study were discarded. Some of the papers returned from our search protocol were not related to the application of blockchain in healthcare industry and were removed. In situations where the relevance of the paper could not easily be determined from the title, the paper was passed to the next stage for further screening. The second phase of the screening involved the reading of the abstracts of the papers that passed through the first stage. In some cases, it was necessary to also read the introduction and the conclusion of a paper to determine if the paper passed our exclusion criteria. 

Our exclusion criteria required us to discard the following: (1) papers that are not peer-reviewed, such as interviews and press announcements; (2) papers without full text availability; (3) papers whose main focus are not related to the application of blockchain technology in healthcare; (4) duplicate papers; (5) papers that are not written in English; (6) retracted papers. Papers that passed these exclusion criteria and that were deemed to be focusing on the application of blockchain in healthcare were included in the next step of the mapping study process.

### 3.4. Keywording on the Basis of the Abstract

**Figure 4:**
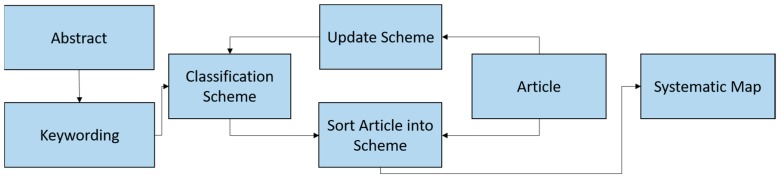
Paper classification process.

This process step was intended to map the relevant research papers in the literature into categories. In doing this, we followed the process described in [19] as depicted in Figure 4. The process involves extracting from the abstracts of the papers some keywords and concepts that reflect the contributions of the papers. Based on these keywords, the papers were clustered into different categories. After clustering the papers into the different categories, each paper was read in detail and if the contents of the paper revealed that it belongs to a different category, the categories were updated. Occasionally, a new category was created if was is observed that the paper does not fit into any of the existing categories. The end result of this process is a mapping of all the relevant papers into a number of different categories.

### 3.5. Data Extraction and Mapping Process

In this last stage of systematic mapping process, information was extracted from the research papers for meta-analysis and to address the research questions. A total of 10 data items were extracted from each paper as shown in Table 2. The first seven items extracted the basic information about the paper, which include the year of publication of the paper, the title, the author(s), the country from where the paper is written (for authors from multiple countries, the country of the corresponding author, or the first author is used), the publication venue, etc. The other data items from 8 to 10 were extracted after the papers were read in detail. In addition to these data items, each of the selected papers was given a number from 1 to 65, which served as the identifier of the paper. The extracted data were collected into Excel sheet for ease of organization and analysis. 

## 4. Results

**Figure 5:**
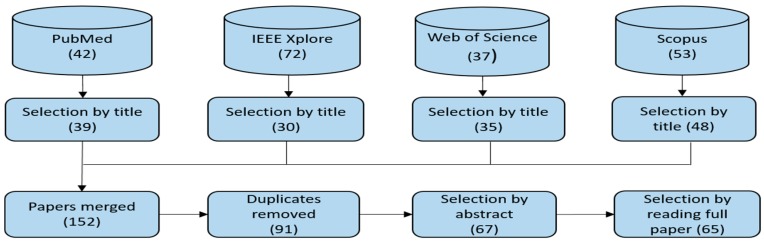
Selection process for the relevant papers.

In this Section, the results of the systematic review are presented. Using our search protocol, we were able to retrieve a total of 204 papers from the scientific databases as shown in Figure 5. After the first screening, which is based on the titles of the papers, 52 papers were excluded, leaving 152 papers for further screening. The papers that were excluded were those that are not related to healthcare; however, healthcare may have been mentioned in their abstracts as one of the non-financial use cases of blockchain, which is why our search protocol retrieved them. Going further, we removed the duplicates by merging the 152 papers in Mendeley, and this reduced the selected papers to 91. In the next screening step, we read the abstracts of the selected papers and in some cases the introduction and the conclusion to further screen the papers in accordance with the criteria defined in Section 3.3. This resulted in the selection of 67 papers. After reading all the selected papers in full, two more papers were excluded for not being focused on healthcare. These papers just have healthcare mentioned in one of the subsections as a potential area of application of blockchain without contributing any new ideas or concepts. At the end of the screening process, there were 65 papers that were selected for inclusion in the study. The full list of the selected papers and some of the extracted data items are included in Table 3. 

Further analysis of the information extracted from these papers is presented in two parts. In the first part, we show the results that are related to the basic information of the papers. In the second part, we discuss the classification and mapping of the relevant papers into different categories.

### 4.1. Basic Information about the Papers

This Section discuss the results related to the first six data items that were extracted from the research papers (Table 2).

#### 4.1.1. Publication Year, Source and Geographical Distribution

**Figure 6:**
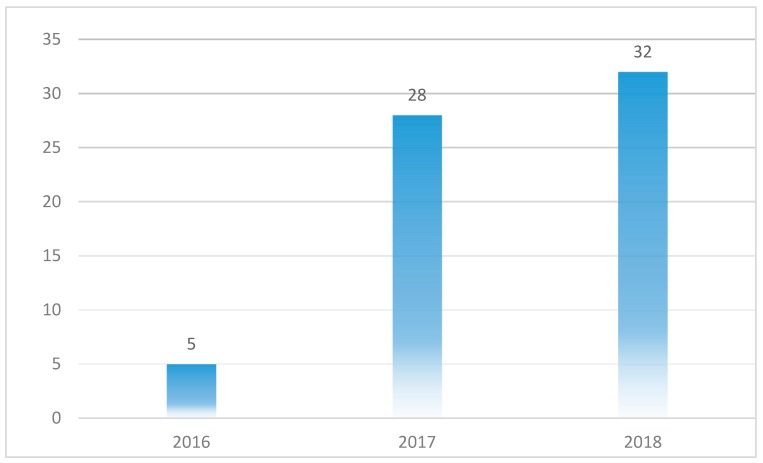
Publication years of the papers.

Even though there was no time limit in our search protocol, all the selected papers were published after the year 2015. Figure 6 shows the distribution of the selected papers, with majority of the selected papers (32% or 49%) published in 2018. Of the 65 selected papers, 28 (43%) were published in 2017 while only five (8%) were published in 2016. What this shows is that research in the area of blockchain application in healthcare is very current and interest in this field is growing rapidly as the number of publications have continued to increase steadily since 2016. 

**Figure 7:**
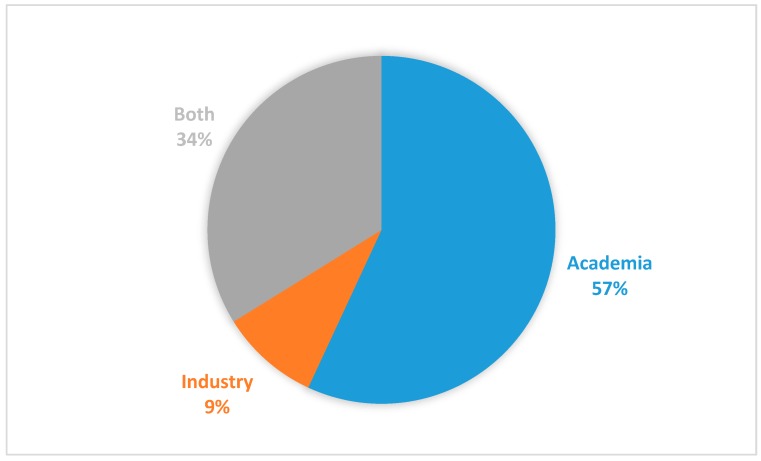
Sources of the papers.

We also show the sources of the selected papers in Figure 7. The source of a paper was used to indicate whether the authors of the paper are affiliated to the academia, the industry or both. As depicted in Figure 7, the majority of the selected papers, 37 (57%) were written by authors that work in different academic institutions, while a paltry 9% (6 papers) were from the industry. The remaining 34% (22 papers) were products of collaborations between industries and the academia. What this shows is that since this technology is still maturing, that industrial players may still be reluctant to start adopting the technology into their operation. However, it should be noted that research conducted (or products developed) in the industry are not usually published in peer-reviewed scientific conferences or journals. Instead, they are published mostly as white papers which were excluded by our search protocols. 

**Figure 8:**
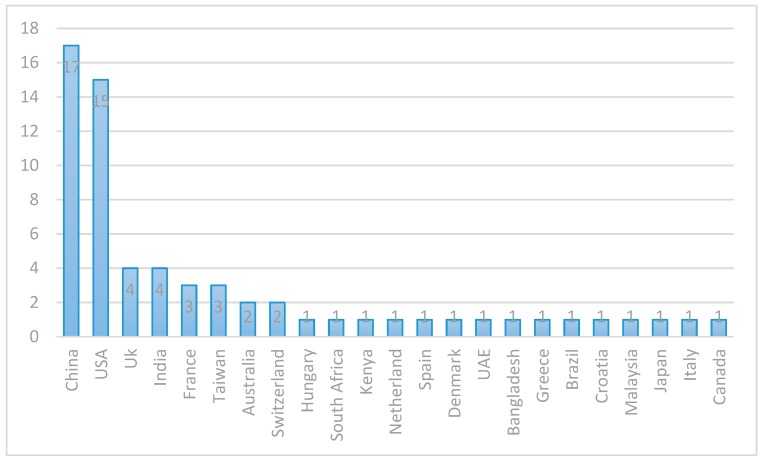
Distribution of the papers by the countries of the authors.

Lastly, to get the idea of the geographical distribution of the members of the research community involved in the research on blockchain application in healthcare, we used the locations (countries) of the institutions the authors of the selected papers are affiliated to. In cases where the authors of a paper are from different countries, we used the country of the corresponding author, or the first author if the corresponding author is unknown. Figure 8 shows the geographic distribution of the research that we selected for this review. The majority of the papers were published by authors in China and USA. China and USA account for 26% and 23% of the selected papers respectively. They are distantly followed by UK and India which account for 6% (four papers) each. France and Taiwan published three papers each, while each of Australia and Switzerland published two papers. Only one paper is published from each of the remaining countries. The distribution shows that 23 countries have published papers on this subject which is indicative of the fact that the application of blockchain in healthcare is gaining global interest. 

#### 4.1.2. Publication Type and Channel

**Figure 9:**
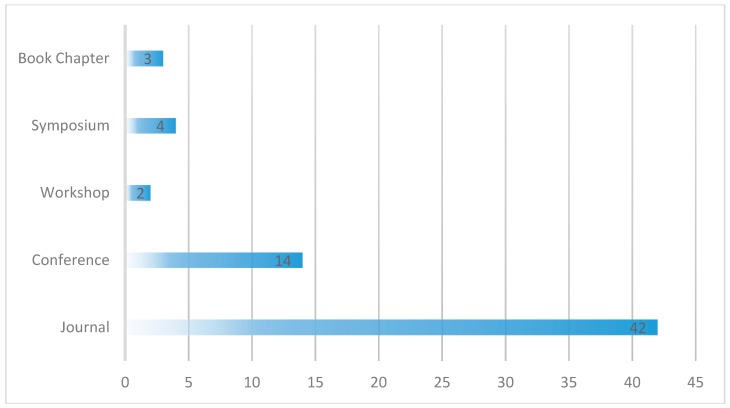
Publication types of the selected papers.

The publication type defines the medium of publication, i.e., whether the paper is published in a journal, conference, symposium, workshop or book chapter. Figure 9 shows the publication types of the selected papers. More than half of the papers selected for this review were published in journals (42 or 65%), 14 of the 65 papers, about 22% were published in conference proceedings, four (6%) were published in Symposiums, three (5%) as book chapters and two (3%) in workshops. The specific publication channels for each of these papers is shown in Table 4. This information could be useful to researchers in finding the right channel to publish their research and to know the conferences and journals to search for the latest research in this field. The 65 selected papers were published in 48 different channels. The majority of the papers were published in the Journal of Medical Systems, followed by IEEE Access where 10 (15%) and four (6%) of the papers were published respectively.

### 4.2. Classification of the Selected Papers

In this Section, we present the results of the classification of the selected papers and the analysis of the extracted data items 8 to 10 of Table 2. After reading all the papers and building the classification scheme as explained in Section 3, it was observed that there are two broad categories of paper types involved in this review which we refer to as reports and technical papers. 

Reports are those papers that lack concrete technical contributions, such as proof-of-concept implementations or rigorous validation procedures. They are mostly secondary publications, such as reviews [13,14,15,16,17,31,55,74], that report previously known solutions, examples, concepts or ideas in relation to the application of blockchain in healthcare. Of the 65 papers included in this review, 19 (29%) are reports. 

The technical papers, on the other hand, are those papers that provide concrete technical contributions to the application of blockchain in healthcare. These include papers that propose new architectures, frameworks or models, or improvements to existing solutions in respect to the application of blockchain in healthcare. Among the technical papers, interest is paid to papers that present or discuss blockchain-based prototype applications that have been developed to solve healthcare problems.

**Figure 10:**
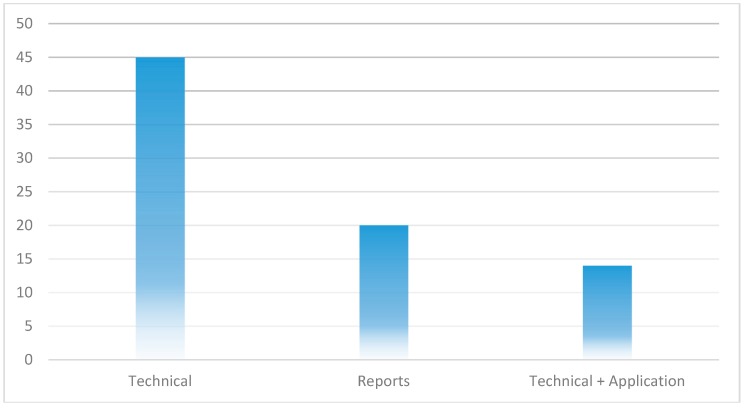
Selected paper types.

**Figure 11:**
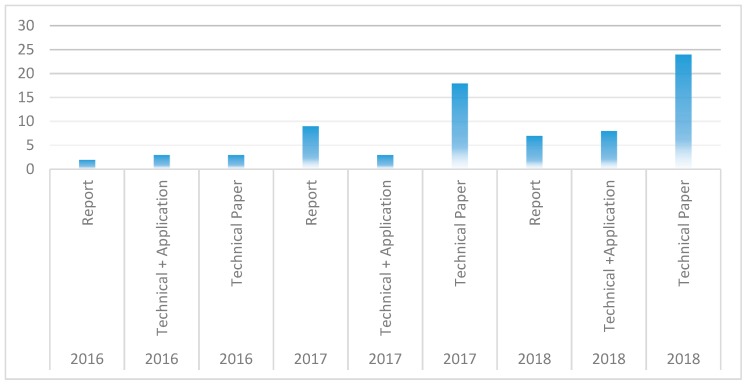
Publication size per paper type per year.

In Figure 10, we show the proportion of the selected papers that are reports, technical papers, and the technical papers that report on developed blockchain-based applications. It can be seen that 69% of the selected papers are technical papers, while about 21% are reports. Out of the 45 papers that are technical, 14 of them (31%) present a proof-of-concept implementation of blockchain-based healthcare application. These papers give insights on how blockchain applications can be developed but the example implementations in the literature are not sufficient and more work needs to be done to bring the blockchain technology to maturity, by developing more applications so that the utility of blockchain in healthcare can be more properly understood and characterized. We also show in Figure 11 how many of the three paper types are published each year, in the three years, from 2016 to 2018. It can be seen that focus on technical papers has been growing progressively as well as the development of blockchain-based healthcare applications.

Reading through the papers, we were able to identify the blockchain use cases in healthcare, some example applications, and the challenges or limitations of developing blockchain-based healthcare solutions. Table 5 gives a summary of the identified use cases and the example applications that have been developed for the respective use cases. 

**Figure 12:**
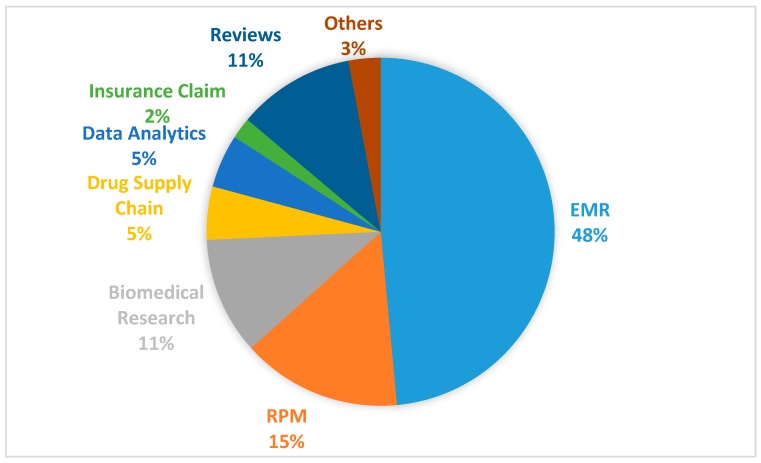
Percentage distribution of the selected papers.

Since each paper seems to address one or more aspects of the identified blockchain use cases in healthcare, we decided to use the identified use cases to further classify the papers. As shown in Figure 12, of the 65 selected papers, seven (11%) of them are review articles in which some of the identified use cases are described. Outside these review articles, 32 (48%) of the papers address specifically the use of blockchain for the management of electronic medical records (EMR), while 10 (15%) of the papers address the application of blockchain in remote patient monitoring (RPM) systems. The remainder of the papers are classified into the following use cases: biomedical research and education (seven papers or 11%)), drugs and pharmaceutical supply chain (three papers or 5%), health data analytics (three papers or 5%), health insurance claim (one paper or 2%) and others (two papers or 3%). In the following subsections, these identified blockchain use cases are discussed in more detail. 

#### 4.2.1. Electronic Medical Record (EMR)

One of the popular use cases of blockchain in healthcare is the management of electronic medical records (EMRs). EMRs, which are sometimes used interchangeably with electronic health records (EHRs) or personal health records (PHRs), have to do with the electronic creation, storage and management of patients’ personal, medical or health-related data. Indeed, EMR use case of blockchain is a major research topic in the literature, with 48% (32) of the 65 selected papers addressing the topic. Blockchain’s property of decentralization, immutability, data provenance, reliability, robustness, the smart contracts, security and privacy are being canvassed as the features that make it very suitable for storage and management of patients’ electronic medical records (EMR) [16,17,33,36,53,74]. Some of the papers are focused on how to facilitate patient-centric data sharing among different healthcare stakeholders, such as providers, researchers and insurers. Consistent with the European General Data Protection Regulation (GDPR) which prohibits the processing of sensitive personal data of patients unless explicit consent is given by the patients [26], blockchain is widely proposed as a viable technology to build the healthcare platform that can empower patients to be in control of how their data are shared, processed or used [13,14,15,24,27,40,45,72,76].

Guardtime, a company that uses a blockchain-based platform to secure over 1 million patients records in Estonia is cited in other reviews as a popular example of the use of blockchain for the management of EMR [13,15]. Another such example is the MedRec project [67], a project of MIT Media Lab and Beth Israel Deaconess Medical Center, which aims at giving patients agency over their own data, to determine who can access them, through some fine-grained access permissions built on blockchain. The Gem Health Network (GHN) is yet another example, which is developed by the US startup, Gem, using the Ethereum blockchain platform. GHN allows different healthcare practitioners to have shared access to the same data [67]. Healthbank, a Swiss digital health company, is similarly working on empowering patients to be in full control of their data using blockchain platform [67]. In [14], the author discusses the Medicalchain project, whose blockchain-based platform will facilitate the sharing of patients’ medical records across international healthcare institutions, and the Healthcoin initiative, which aims at constructing a global EMR system. Other players working on different initiatives and projects based on blockchain-enabled patient-centric EMR include Factom, HealthCombix, Patientory, SimplyVital, IBM’s Watson, BurstIQ, Bowhead, QBRICS and Nuco [14].

Some of the barriers to blockchain-enabled patient-centric electronic medical records include interoperability among disparate blockchain-based EMR solutions (because of lack of standards), scalability (high volume of clinical data), patient engagement (not all patients are willing and able to manage their own data), data security and privacy, and lack of incentives [14,24,40,44,55,66,84].

Some workarounds have been proposed to tackle some of these challenges. For example, as a countermeasure to the challenge of scalability, given the large volume of clinical data involved, the trend is to store the actual healthcare data on the cloud and store only the pointers to the data on blockchain, along with their fingerprints [30,35,45]. A good number of the technical papers report on the implementation of blockchain-based EMR applications in which different approaches are adopted to address these challenges. Yet, some publications propose different solutions to improve the security and privacy of the EMR data on blockchain. 

HealthChain [42] is an EMR application developed as a permissioned, private blockchain network using the IBM Blockchain’s Hyperledger Fabric and deployed on Bluemix. The modular architecture of Hyperledger Fabric [12] enables HealthChain to achieve health data confidentiality, scalability and security. HealthChain also incorporates chaincodes (smart contracts) that control authorizations and access privileges on the blockchain network. There is also another blockchain-based framework, Ancile [34], which similarly utilizes smart contracts, but is built on Etherum blockchain platform to achieve access control, data security, privacy and interoperability of electronic medical records. MedRec [67] (earlier discussed) and the medical data preservation system (DPS) developed by Li et al. [46] are two other examples of blockchain implementations of EMR that utilize the Ethereum blockchain platform. Other blockchain-based EMR applications include MedBlock [64], BlockHIE [48], FHIRChain [54] and MeDShare [68].

In the area of security and privacy of the sensitive data stored on blockchain-based EMR, some cryptographic schemes are proposed to strengthen the security and validity of the EMRs stored on the blockchain. Hussein et al. [28] propose a blockchain-based access control method to EMR that employs Discrete Wavelength Transform and Genetic Algorithm to enhance the security and optimize the performance of the system. An attribute-based signature scheme with multiple authorities is also proposed, in which the patient is able to endorse a message to be added to the blockchain based on attributes of the message, without disclosing any sensitive information. This protocol is shown to resist collusion attack and is demonstrated to be computationally secure [77]. In a similar way, an attribute-based encryption (ABE), identity-based encryption (IBE) and identity-based signature (IBS) are proposed to be used with blockchain in [78]. Other security-related proposals for blockchain-based EMR include the key management schemes by Zhao et al. [52,63]. Zhang and Poslad [39] propose an architecture called GAA-FQ (Granular Access Authorization supporting Flexible Queries) which provides secure authorization at different levels of granularity without requiring public key infrastructure (PKI).

Shifting emphasis to privacy, Zhang and Lin [83] propose a blockchain-based secure and privacy-preserving EMR scheme which uses private and consortium blockchains to store the actual EMR and the pointers to the EMR respectively. This scheme also relies on asymmetric encryption but also implements mechanisms for conformance testing to ensure the system’s availability. In [65], the authors propose a privacy-preserving platform, MediBchain that employs cryptographic functions to deidentify patients’ data in blockchain-based EMR systems. Yeu et al. in [58] also propose an architecture called Healthcare Data Gateway (HDG) for blockchain-based EMR application which allows patients to own, control and choose how to share their data in a privacy-preserving manner. A related architecture is proposed for the management and sharing of medical data of diabetes patients using multi-signature blockchain contracts to achieve access control and data privacy [59]. 

#### 4.2.2. Drug/Pharmaceutical Supply Chain

One other identified use case of blockchain is in health supply chain management, particularly in the drug/pharmaceutical industry. The delivery of counterfeit or substandard medications can have dire consequences for the patients, yet this is a common problem faced in the pharmaceutical industry. Blockchain technology has been identified as having the capability to address this problem [13,15,55,56,74]. 

Engelhardt, in his survey, mentions some companies that are working on how blockchain can be used to detect prescription drug fraud. The companies mentioned include Nuco, HealthChainRx and Scalamed [14]. The general idea is to record every transaction relating to the prescription of drugs on the blokchain network to which all the stakeholders (manufacturers, distributors, doctors, patients and pharmacists) are connected. This way, any alteration or malicious modification of the prescription by any of the parties can be detected. Mettler also mentions the Counterfeit Medicines Project that is launched by Hyperledger (the developers of Hyperledger Fabric [12]) to combat drug counterfeiting. 

Only one paper in this review presents an implementation of an example blockchain-based application for pharmaceutical supply chain management. Modum.io AG is a startup that uses blockchain to achieve data immutability while creating public accessibility of the temperature records of pharmaceutical products during their transportation so that their compliance to quality control temperature requirements can be verified [47]. Mackey and Nayyar, however, report that they found from grey literature many examples of prototypes and research initiatives related to the application of blockchain in the area of pharmaceutical supply chain management [31]. This indicates that industrial players may have released many commercial blockchain-based products to combat the fake medicine trade even when there are still limited academic publications on the subject.

#### 4.2.3. Biomedical Research and Education

Blockchain has an interesting use case in biomedical research and education. In clinical trials, blockchain can help to eliminate falsification of data and the under-reporting or exclusion of undesirable results of clinical research [41,73,74,82]. Blockchain makes it easier for patients to grant permission for their data to be used for clinical trials because of the anonymization that is inherently encoded in the data [55]. Additionally, the immutability property of blockchain certifies the integrity of data collected through blockchain for clinical study. The transparent and public nature of blockchain also make it easier to replicate research from blockchain-based data. All these are some of the reasons blockchain is expected to revolutionize biomedical research [14,17]. Blockchain has also been noted to have the potential to revolutionize the peer-review process for clinical research publications based on its decentralized, immutable and transparent properties [17]. Another potential application of blockchain to health professions education (HPE) is presented in [43] where Funk et al. make a case for using blockchain to build an HPE system that will be value-based, competency-based and offer credentialing services without relying on a third-party. 

A proof of concept implementation of consent traceability in clinical trial using blockchain protocol is presented in [38]. Similarly, Nugent et al. present their research in [60] in which they demonstrate how smart contracts on Ethereum blockchain platform can be used to improve data transparency in clinical trials. The Ethereum platform is also used to implement another blockchain-based solution that is proposed to notarize documents retrieved from biomedical databases [71].

#### 4.2.4. Remote Patient Monitoring (RPM)

In this Section, we look at how blockchain technology facilitates remote patient monitoring (RPM). Remote patient monitoring involves the collection of biomedical data through body area sensors (or IoT devices) and mobile devices to be able to remotely monitor the status of the patient outside traditional healthcare environments such as the hospital. Blockchain has been proposed as a means for storing, sharing and retrieving the remotely-collected biomedical data [29,32,37]. 

In [57], Griggs et al. demonstrate how smart contracts on the Ethereum blockchain platform can support real-time patient monitoring application with capability to provide automated interventions in a secure environment. Liang et al. [61] present a Hyperledger-based implementation of blockchain-enabled data collection and sharing among healthcare stakeholders in a mobile healthcare environment. Similarly, blockchain is employed to develop SMEAD, mobile-enabled assisting device for monitoring diabetes patients [79]. Another example application is presented in [81] where mobile devices (smartphones) were successfully used to transmit data to a blockchain-based application on Hyperledger Fabric. Ashraf Uddin et al. also developed a blockchain-based patient centric agent (PCA) to achieve end-to-end data security and privacy in a continuous remote patient monitoring application [50]. In [75], the authors proposed to use practical swarm optimization (PSO) for root exploit detection and feature optimization in blockchain-based mobile device medical data management. Lastly, Ji et al. proposed a scheme known as BMPLS (Blockchain-based Multi-level Privacy-preserving Location Sharing) for realizing privacy-preserving location sharing for remote monitoring applications. 

#### 4.2.5. Health Insurance Claims

Insurance claims processing in healthcare can benefit from blockchain’s transparency, decentralization, immutability and auditability of records stored on it [55]. A number of papers identify insurance claim processing as a very promising area for the application of blockchain in healthcare [13,17,40,55,86]. However, examples of prototype implementations of such systems are very limited. One good example we can find is the MIStore (a blockchain-based medical insurance storage system) which is deployed on the Ethereum blockchain platform [70]. Additionally, [14] talks about an initiative by a company named Pokitdok that aims to partner with Intel to build a blockchain-based system that will facilitate insurance claim resolution in healthcare.

#### 4.2.6. Health Data Analytics (HDA)

Blockchain provides also a unique opportunity to harness the power of other emerging technologies such as deep learning and transfer learning techniques to realize predictive analytics of healthcare data and advance the research in the area of precision medicine [85]. This blockchain use case is also mentioned in [55] and [17], while [51] provides a comprehensive roadmap on how this can be realized. Juneja and Marefat conducted an experimental research in which blockchain is used in a deep-learning architecture for arrhythmia classification [62].

#### 4.2.7. Others

There are other potential areas of application of blockchain in healthcare, including areas such as the dental industry, legal medicine and meaningful use [14,17], but there are no papers in our review that address such use cases. On the contrary, there are two papers that cannot be classified under any of the identified use cases of blockchain, but they present relevant research perspectives. One sets out to identify the metrics for evaluating blockchain-based healthcare applications [69] while the other [80] studies the socio-technical implications of using blockchain technology in healthcare.

## 5. Discussion

We use this Section to cross-examine the research questions posed by this research in the light of the results of the study. We also discuss the limitations of the study and its validity. 

### 5.1. What Are the Use Cases of Blockchain in Healthcare?

From the mapping study, the blockchain use cases in healthcare include the managements of electronic medical records (EMRs), pharmaceutical supply chain, biomedical research and education, remote patient monitoring (RPM), health insurance claims, health data analytics and other potential areas of healthcare applications. 

Based on the selected papers, a substantial portion of the research (48%) concentrates on the application of blockchain in the management of electronic medical records. Traditionally, patients’ records are stored separately in different databases across different service providers, with little or no interoperability. This leaves the control of the health data mostly in the hands of the service providers and also limits the collaborative sharing of such data among healthcare stakeholders. By applying blockchain to the management of EMRs, patients will be in control of their own health data and be able to decide how they are used. Data sharing between healthcare stakeholders will be easier, better controlled, transparent and trustworthy. However, using blockchain to store EMRs brings up concerns about the security and privacy of patients’ sensitive information, an issue that a good portion of the research is also dedicated to, e.g., [28]. 

Some companies and research projects, such as Guardtime [13] and MedRec [67] have developed blockchain-based EMR applications. In addition, a number of the reviewed materials discuss the prototype implementations of different blockchain-based EMR applications [48,54,64,68]. 

Similarly, blockchain use cases in pharmaceutical supply chain management, biomedical research/education and remote patient monitoring have received considerable research attention with example prototype implementations (see Table 5). There is also one example implementation of prototype application relating to health insurance claim processing [70]. However, other use cases are still mostly at the conceptual level. 

**Figure 13:**
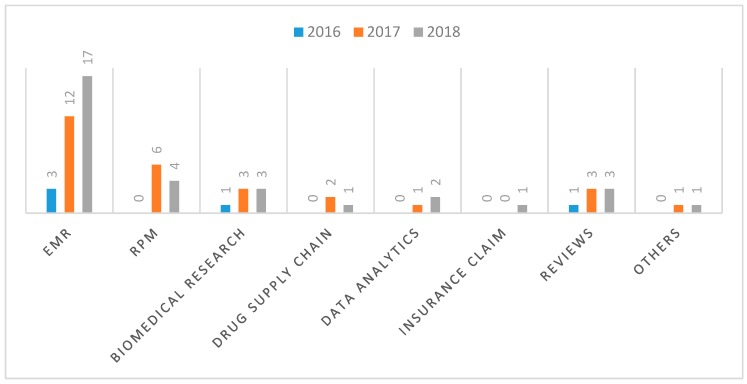
Classification of the selected papers showing publication trend from 2016 to 2018.

To identify the research trends as it concerns the identified use cases of blockchain in healthcare, the diagram of Figure 11 was extended as shown in Figure 13 to further highlight the number of papers published for the respective use cases from 2016 to 2018. 

### 5.2. Of the Identified Use Cases, What Blockchain-Based Applications Have Been Developed?

In general, the technology is still maturing and even when prototype applications are developed, in some cases, they are just for experimental purposes with very basic functionalities. That said, there are papers that present implementation details of applications that have been developed for the various use cases. 

For the EMR use case, example applications include the Healthchain [42] which is developed on Hyperledger Fabric, Acile [34] and MedRec [67], both of which are developed on the Ethereum platform. Other examples include MedBlock [64], BlockHIE [48], FHIRChain [54] and MeDShare [68]. 

Similarly, other blockchain use cases, such as the management of the drug/pharmaceutical supply chain, biomedical research and education, health insurance claim processing and remote patient monitoring have example use cases as discussed in Section 4.2. There are, however, some potential use cases of blockchain that are still at the conceptual levels where prototypes have not yet been developed, such as the application of blockchain in legal medicine [17]. Table 5 gives a summary of the blockchain use cases in healthcare and the corresponding examples of prototype applications that have been developed for the respective use cases. 

It is also interesting to observe that most of these applications are developed on popular blockchain frameworks, such as Ethereum and Hyperledger Fabric as shown in Table 6.

### 5.3. What Are the Challenges and Limitations of the Blockchain-Based Applications?

Some identified challenges to the development of blockchain-based applications include interoperability, security and privacy, scalability, speed and patient engagement [55]. 

The interoperability challenge stems from the fact that there is not yet an existing standard for developing blockchain-based healthcare applications; therefore, applications developed by different vendors or on different platforms may not be able to interoperate. Consider, for example, the two remote patient monitoring applications, in which one is developed on the Ethereum platform [57] while the other is developed on the Hyperledger Fabric platform [61] (see Table 6), it would be difficult to exchange information from one platform to the other.

With regards to the security and privacy of blockchain-based healthcare applications, there is a concern that despite the encryption techniques employed, it could still be possible to reveal the identity of a patient in a public blockchain by linking together sufficient data that are associated to that patient [74]. In addition, there is also the potential risk of security breaches that could arise from intentional malicious attacks to the healthcare blockchain by criminal organizations or even government agencies that could compromise the privacy of the patients. There have been several cases of reported attacks on the blockchain networks that power different cryptocurrencies [21]. The private keys that are used for data encryption and decryption in blockchain are also prone to potential compromise which could result in unauthorized access to the stored health data. 

Furthermore, there is the concern that the immutability property of blockchain does not augur well with the GDPR’s “right to be forgotten,” which is part of the European Union General Data Protection Regulation which stipulates that the user has the right to request for the complete erasure of the user’s data [26]. Since the immutability of blockchain ensures that data once saved to the blockchain cannot be deleted or altered, it could prove counterproductive when it is desirable to completely wipe out the medical history of a patient. 

Scalability of blockchain-based healthcare solutions is a major challenge especially in relation to the volume of data involved. It is not optimal, or even practicable in some cases, to store the high-volume biomedical data on blockchain as this is bound to cause serious performance degradation. There is also the problem of speed as the blockchain-based processing can introduce some significant latency. For example, the validation mechanism in the current set-up of the Ethereum blockchain platform necessitates all the nodes in a network to participate in the validation process [21]. This incurs considerable processing delay, especially if the data load is significant. 

One more challenge is how to engage patients in the management of their data on blockchain. Patients, especially the elderly and the young, may not be interested or able to participate in the management of their health data [74].

### 5.4. How Are These Challenges and Limitations Currently Being Addressed?

Some workarounds are being proposed to circumvent some of the challenges and limitations posed by the application of blockchain in health IT (information technology) systems. For example, as a countermeasure to the problem of scalability, it is proposed to store the encrypted health data “off-chain,” such that only some condensed information about the data and how they can be accessed are stored on the blockchain [35,40,45]. This also takes care of the GDPR’s “right to be forgotten” problem, since the actual health data stored off-chain can be permanently deleted, even if the pointer to the data on the blockchain cannot be deleted. However, this countermeasure has some limitations such as the fact that the redundancy that is built into blockchain, which enhances data availability, is partially lost. 

To further secure the data and protect patients’ privacy, permissioned blockchain such as the private or consortium blockchain is used instead of the permissionless, public blockchain for healthcare applications [42]. In addition, by following rigorous software development process and applying all known security measures during code development, much of the security threats may be contained. In permissioned healthcare blockchains, controls are also put in place to be able to reverse fraudulent or invalid transactions [24]. With the blockchain-based smart contracts [2], different rules can be defined and programmed to control how the healthcare application behaves and how it handles the patients’ data. 

Furthermore, to improve the performance of the system and enhance the processing speed, only some nodes are permitted to participate in the consensus and validation processes [42]. This is in contrast to the protocols in public blockchain, such as Bitcoin, in which any node can take part in the consensus or validation process. 

### 5.5. What Are the Open Research Issues and the Areas for Future Research?

As the blockchain technology application in healthcare is still an emerging field, there is need for researchers to develop more prototypes and proof-of-concepts to deepen the understanding and maturity of the technology in relation to its application in healthcare. Many of the proposed frameworks, concepts, models and architectures, such as [51], need to be implemented and tested to evaluate their strengths and weaknesses. 

To guarantee interoperability between different blockchain products, there is need for open standards. Currently, the focus is on testing the functionality of blockchain prototypes for proof of concepts. However, for blockchain to be fully adopted and deployed in operational healthcare environments, open standards for interoperability need to be defined. It is therefore important that researchers start looking into the interoperability issues and the standardization processes. There is already a standards group (ISO/TC 307) to which researchers can send in their contributions [14]. 

The challenges of data security and privacy, interoperability, scalability and speed that characterize blockchain-based healthcare applications are all open research issues that require concerted further research engagements in order to improve stakeholders’ confidence in the use of the technology and to foster its adoption in healthcare. 

### 5.6. Limitations of the Study

Systematic mapping studies can be marred by publication or selection bias, errors in data extraction or miscalculations [21].

Publication bias arises from the tendency for researchers to publish more positive results than negative ones because positive results are more likely to be accepted for publication and also more likely to be cited by others. It is difficult to overcome publication bias from the perspective of the reviewers, however; we made efforts to address this by searching different but reputable scientific databases to retrieve as many relevant publications as possible. We were thus able to find many papers, and our chances of retrieving any publications with negative results were significantly enhanced. However, by searching only peer-reviewed articles and excluding grey literature in our search protocol, we stand a high chance of missing some important grey publications such as white papers from industries. We believe, however, that by using only scientific databases to search for peer-reviewed articles, we have more chances of retrieving high quality scientific publications.

Selection bias, on its part is more controllable by the reviewers since it has to do with the tendency to exclude some relevant publications in the analysis by using a flawed search protocol. In our case, we took the time to design a search protocol and our analysis showed that our search protocol was able to retrieve every relevant paper. In defining our inclusion and exclusion criteria, care was also taken to ensure that the selected papers would represent an unbiased sampling of all the papers that were relevant to our research. However, as earlier stated, the fact that we based our research only on peer-reviewed publications means that we were not able to access, for example, information published on company websites, discussion forums and other such related venues. Interestingly, we realize that most of the important information available in those grey literatures, like the different blockchain-based healthcare applications, has been synthesized and put into some peer-reviewed publications. For example, [14] has a list of a number of important blockchain applications, some of which we are not able to retrieve directly by using our search protocol. Therefore, through such secondary publications, we were able to compensate for the information we may have missed by not searching the grey literature.

Errors in data extraction and miscalculations could stem from the inability of the reviewers to accurately and properly extract information and data from the selected papers. To address this limitation, we made use of a reference management software, Mendeley, to organize and manage all the papers we downloaded in relation to this study. We further employed Excel for recording and organizing the extracted data items, as well as for performing statistical analysis on the extracted data, while also being painstakingly meticulous and rigorous in our analysis to avoid introducing any human errors. 

## 6. Conclusions

Blockchain technology has evolved from the time it was introduced to the world through Bitcoin into a general-purpose technology with use cases in many industries including healthcare. To understand the state-of-the-art of the application of blockchain technology in healthcare, we conducted a systematic review in which we created the map of all relevant research using the systematic mapping study process [19]. Specifically, the objectives of the study were to identify the blockchain technology use cases in healthcare, the example applications that have been developed for these use cases, the challenges and limitations of the blockchain-based healthcare applications, the current approaches employed in developing these applications and areas for future research. Our search and paper selection protocol produced 65 papers which we analyzed to address the research questions.

Our study shows that blockchain has many healthcare use cases including the management of electronic medical records, drugs and pharmaceutical supply chain management, biomedical research and education, remote patient monitoring, health data analytics, among others. A number of blockchain-based healthcare applications have been developed as prototypes based on emerging blockchain paradigms, such as smart contracts, permissioned blockchain, off-chain storage, etc. However, more research still needs to be conducted to better understand, characterize and evaluate the utility of blockchain technology in healthcare. Further research is also needed to supplement ongoing efforts to address the challenges of scalability, latency, interoperability, security and privacy in relation to the use of blockchain technology in healthcare. 

## Figures and Tables

**Table 1:** Benefits of blockchain to healthcare applications.

Decentralization	The very nature of healthcare, in which there are distributed stakeholders, requires a decentralized management system. Blockchain can become that decentralized health data management backbone from where all the stakeholders can have controlled access to the same health records, without any one playing the role of a central authority over the global health data.
Improved data security and privacy	The immutability property of blockchain greatly improves the security of the health data stored on it, since the data, once saved to the blockchain cannot be corrupted, altered or retrieved. All the health data on blockchain are encrypted, time-stamped and appended in a chronological order. Additionally, health data are saved on blockchain using cryptographic keys which help to protect the identity or the privacy of the patients.
Health data ownership	Patients need to own their data and be in control of how their data is used. Patients need the assurance that their health data are not misused by other stakeholders and should have a means to detect when such misuse occurs. Blockchain helps to meet these requirements through strong cryptographic protocols and well-defined smart contracts.
Availability/robustness	Since the records on blockchain are replicated in multiple nodes, the availability of the health data stored on blockchain is guaranteed as the system is robust and resilient against data losses, data corruption and some security attacks on data availability.
Transparency and trust	Blockchain, through its open and transparent nature, creates an atmosphere of trust around distributed healthcare applications. This facilitates the acceptance of such applications by the healthcare stakeholders.
Data verifiability	Even without accessing the plaintext of the records stored on blockchain, the integrity and validity of those records can be verified. This feature is very useful in areas of healthcare where verification of records is a requirement, such as pharmaceutical supply chain management and insurance claim processing.

**Table 2:** Extracted data items

#	Data Item	Description
1	Year	Publication year of the paper
2	Title	Title of the paper
3	Authors	The authors of the paper
4	Country	Country of affiliation of the authors
5	Publication channel	The channel through which the paper is published
6	Publication type	Journal/Conference/Workshop/etc.
7	Publication source	Academia/Industry/Both
8	Paper type	Type based on classification scheme
9	Paper contributions	Main contributions of the paper
10	Summary	Our own summary or abstract of the paper

**Table 3:** List of selected papers.

#	Authors and Paper Ref.	Year	Publication Type	Use Case
1	Patel V. [27]	2018	Journal	Electronic Medical Records
2	Ahmed F. et al. [28]	2018	Journal	Electronic Medical Records
3	Tushar D. et al. [29]	2017	Conference	Remote Patient Monitoring
4	Kaur H. et al. [30]	2018	Journal	Electronic Medical Records
5	Mackey T. [31]	2017	Journal	Drug/Pharmaceutical Supply Chain
6	Zhang J. [32]	2017	Journal	Remote Patient Monitoring
7	Liu W. [33]	2017	Workshop	Electronic Medical Records
8	Gaby G. et al. [34]	2018	Journal	Electronic Medical Records
9	Xia et al. [35]	2017	Journal	Electronic Medical Records
10	Magyar G. [36]	2017	Workshop	Electronic Medical Records
11	Weiss M. et al. [37]	2017	Conference	Remote Patient Monitoring
12	Kuo T. et al. [16]	2017	Journal	Review
13	Benchoufi M. [38]	2018	Journal	Biomedical Research/Education
14	Zhang X. et al. [39]	2018	Conference	Electronic Medical Records
15	Angraal S. et al. [13]	2017	Journal	Review
16	Gordon W. et al. [40]	2018	Journal	Electronic Medical Records
17	Benchoufi M. [41]	2017	Journal	Biomedical Research/Education
18	Mettler M. et al. [15]	2016	Journal	Review
19	Ahram T. [42]	2017	Journal	Electronic Medical Records
20	Funk E. et al. [43]	2018	Journal	Biomedical Research/Education
21	Kamau G. et al. [44]	2018	Conference	Electronic Medical Records
22	Esposito C. et al. [45]	2018	Journal	Electronic Medical Records
23	Li H. et al. [46]	2018	Journal	Electronic Medical Records
24	Bocek T. [47]	2017	Symposium	Drug/Pharmaceutical Supply Chain
25	Jiang S. [48]	2018	Conference	Electronic Medical Records
26	Ji Y. et al. [49]	2018	Journal	Remote Patient Monitoring
27	Uddin A. et al. [50]	2018	Journal	Remote Patient Monitoring
28	Mamoshina P. et al. [51]	2017	Journal	Health Data Analytics
29	Zhao H. et al. [52]	2018	Journal	Electronic Medical Records
30	Cunningham J. et al. [53]	2017	Journal	Electronic Medical Records
31	Zhanga P. et al. [54]	2018	Journal	Electronic Medical Records
32	Kamel Boulos M. et al. [55]	2018	Journal	Review
33	District N. et al. [56]	2018	Journal	Drug/Pharmaceutical Supply Chain
34	Grggs K. et al. [57]	2018	Journal	Remote Patient Monitoring
35	Yue X. et al. [58]	2016	Journal	Electronic Medical Records
36	Engelhardt M. [14]	2017	Journal	Review
37	Roman-Belmonte et al. [17]	2018	Journal	Review
38	Cichosz S. et al. [59]	2018	Journal	Electronic Medical Records
39	Nugent T. et al. [60]	2016	Journal	Biomedical Research/Education
40	Liang X. et al. [61]	2017	Symposium	Remote Patient Monitoring
41	Alhadhrami Z. et al. [24]	2017	Conference	Electronic Medical Records
42	Marefat M. et al. [62]	2018	Conference	Health Data Analytics
43	Zhao H. et al. [63]	2017	Symposium	Electronic Medical Records
44	Fan K. et al. [64]	2018	Journal	Electronic Medical Records
45	Al Omar A. et al. [65]	2017	Book Chapter	Electronic Medical Records
46	Tak P. et al. [66]	2016	Book Chapter	Electronic Medical Records
47	Azaria A. et al. [67]	2016	Conference	Electronic Medical Records
48	Xia Q et al. [68]	2017	Journal	Electronic Medical Records
49	Zhang P. et al. [69]	2017	Conference	Dapps Evaluation
50	Zhou L. et al. [70]	2018	Journal	Insurance Claim
51	Mytis-Gkometh P. et al. [71]	2018	Book Chapter	Biomedical Research/Education
52	Roehrs A. et al. [72]	2017	Journal	Electronic Medical Records
53	Shae Z. et al. [73]	2017	Conference	Biomedical Research/Education
54	Radanović I. et al. [74]	2018	Journal	Review
55	Firdaus A. et al. [75]	2018	Journal	Remote Patient Monitoring
56	Dubovitskaya A. et al. [76]	2017	Symposium	Electronic Medical Records
57	Guo R. et al. [77]	2018	Journal	Electronic Medical Records
58	Wang H. et al. [78]	2018	Journal	Electronic Medical Records
59	Saravanan M. et al. [79]	2017	Conference	Remote Patient Monitoring
60	Wong M. et al. [80]	2018	Journal	Social-technical Issues
61	Ichikawa D. et al. [81]	2017	Journal	Remote Patient Monitoring
62	Angeletti F. et al. [82]	2017	Conference	Biomedical Research/Education
63	Zhang A. et al. [83]	2018	Journal	Electronic Medical Records
64	Rifi N. et al. [84]	2017	Conference	Electronic Medical Records
65	Shae Z. et al. [85]	2018	Conference	Health Data Analytics

**Table 4:** Publication channels.

Health Informatics Journal	[27]
Cognitive Systems Research	[28]
International Conference on Intelligent Sustainable Systems (ICISS)	[29]
Journal of medical systems	[30,46,49,57,58,64,70,75,78,83]
Expert Opinion on Drug Safety	[31]
IEEE Access	[32,50,68,77]
International Workshop on Emerging Technologies for Pervasive Healthcare and Applications (ETPHA)	[33]
Sustainable Cities and Society	[34]
Information	[35]
Neumann Colloquium (NC)	[36]
IST-Africa Conference	[37,44]
Journal of the American Medical Informatics Association	[16]
F1000Research	[27,60]
IEEE International Conference on Communications (ICC)	[39]
Circulation: Cardiovascular Quality and Outcomes	[13]
Computational and Structural Biotechnology Journal	[40,54]
Trials	[41]
International Conference on e-Health Networking, Applications and Services (Healthcom)	[15,69]
IEEE Technology and Engineering Management Conference (TEMSCON)	[42]
Academic Medicine	[43]
IEEE Cloud Computing	[45]
IEEE International Symposium on Integrated Network Management	[47]
IEEE International Conference on Smart Computing	[48]
Oncotarget	[51]
CAAI Transactions on Intelligence Technology	[52]
Studies in Health Technology and Informatics	[53]
International Journal of Health Geographics	[55]
International journal of environmental research and public health	[56]
Technology Innovation Management Review	[14]
Postgraduate Medicine	[17]
Journal of diabetes science and technology	[59]
IEEE International Symposium on Personal, Indoor, and Mobile Radio Communications (PIMRC)	[61]
IEEE International Conference on Electrical and Computing Technologies and Applications (ICECTA)	[24]
IEEE EMBS International Conference on Biomedical and Health Informatics (BHI)	[62]
IEEE International Symposium on Autonomous Decentralized Systems	[63]
Lecture Notes in Computer Science	[65,66]
IEEE International Conference on Open and Big Data	[67]
Precision Medicine Powered by pHealth and Connected Health	[71]
Journal of Biomedical Informatics	[72]
IEEE International Conference on Distributed Computing Systems	[73,85]
Applied health economics and health policy	[74]
AMIA … Annual Symposium proceedings. AMIA Symposium	[76]
EEE International Conference on Advanced Networks and Telecommunications Systems (ANTS)	[79]
JMIR mHealth and uHealth	[81]
International Conference on Software, Telecommunications and Computer Networks (SOFTCOM)	[82]
International Conference on Advances in Biomedical Engineering (ICABME)	[84]

**Table 5:** Use cases and example applications.

Use Cases	Example Applications
Electronic Medical Records	HealthChain [42], Ancile [34], MedRec [67], DPS [46], MedBlock [64], BlockHIE [48], FHIRChain [54] and MedShare [68]
Drug/Pharmaceutical Supply Chain	Medium.io AG [47]
Biomedical Research and Education	[38,60,71]
Remote Patient Monitoring	[50,57,61,79,81]
Health Insurance Claims	MIStore [70]
Health Data Analytics	[51,62]

**Table 6:** Blockchain frameworks used in developing healthcare applications.

Frameworks	Example Applications
Ethereum	MedRec [67], Ancile [34], DPS [46], GHN [15], FHIRChain [54], MedShare [68], SMEAD [79], Medium.io AG [47], MIStore [57,60,70,71]
Hyperledger Fabric	HealthChain [42], MedicalChain [40,61,62,81]
Bitcoin	[38]
Proprietary	Guardtime [13], MedBlock [64], BlockHIE [48,50]
